# Geospatial distribution and bypassing health facilities among National Health Insurance Scheme enrollees: implications for universal health coverage in Nigeria

**DOI:** 10.1093/inthealth/ihab039

**Published:** 2021-06-29

**Authors:** David A Adewole, Steve Reid, Tolu Oni, Ayo S Adebowale

**Affiliations:** Department of Health Policy and Management, Faculty of Public Health, College of Medicine, University of Ibadan, Ibadan, Nigeria; Department of Public Health & Family Medicine, Division of Public Health Medicine, University of Cape Town, Cape Town, South Africa; Primary Health Care Directorate, Faculty of Health Sciences, University of Cape Town, E47 OMB Groote Schuur Hospital, Observatory, Cape Town 7925, South Africa; Medical Research Council Epidemiology Unit, University of Cambridge, Cambridge, UK; Division of Public Health Medicine, School of Public Health and Family Medicine, University of Cape Town, Cape Town, South Africa; Department of Epidemiology and Medical Statistics, Faculty of Public Health, University of Ibadan, Ibadan, Nigeria

**Keywords:** bypassing, geospatial mapping, health insurance, healthcare access, Nigeria, universal health coverage

## Abstract

**Background:**

This study was carried out to enable an assessment of geospatial distribution and access to healthcare facilities under the National Health Insurance Scheme (NHIS) of Nigeria. The findings will be useful for efficient planning and equitable distribution of healthcare resources.

**Methods:**

Data, including the distribution of selected health facilities, were collected in Ibadan, Nigeria. The location of all facilities was recorded using Global Positioning System and was subsequently mapped using ArcGIS software to produce spider-web diagrams displaying the spatial distribution of all health facilities.

**Results:**

The result of clustering analysis of health facilities shows that there is a statistically significant hotspot of health facility at 99% confidence located around the urban areas of Ibadan. The significant hotspot result is dominated by a feature with a high value and is surrounded by other features also with high values. Away from the urban built-up area of Ibadan, health facility clustering is not statistically significant. There was also a high level (94%) of bypassing of NHIS-accredited facilities among the enrollees.

**Conclusions:**

Lopsided distribution of health facilities in the study area should be corrected as this may result in inequity of access to available health services.

## Introduction

The magnitude and nature of determinants of access to healthcare differ between and within countries.^1,2^ This also determines the inequity of access to healthcare and thus difficulties in achieving universal health coverage (UHC).^3^ Access to care has been discussed along with spatial availability and geographical accessibility to healthcare facilities, among other dimensions.^4^ Geographical accessibility defines the extent to which distant facilities are patronised, especially in rural areas where healthcare facilities are inadequate in number and are also more spread out.^4,5^ Globally, problems of spatial availability and geographical accessibility to healthcare facilities are some of the health system's identified challenges.^6^ Health outcomes are poor in Nigeria: life expectancy at birth is 54 y, birth attended by skilled health staff is 43% of the total and the under 5 mortality rate is 120 per 1000 live births.^7^ The poor performance of the health system in Nigeria has been attributed to inequity of physical access to available health facilities among other factors.^5,8^

The National Health Insurance Scheme (NHIS) was established about 15 y ago to reduce inequity of access to care.^9^ However, studies that have been conducted about the NHIS have focused primarily on the financial function and related factors of inequity of access to the health system.^10^ Although the primary healthcare (PHC) level is the officially recognised point of entry into the health system in Nigeria, under the NHIS, only the secondary and the tertiary levels of care are used for service provision.^11^ To the best of our knowledge, other factors with enormous impact on access to care, such as availability and geospatial distribution of available healthcare facilities under the NHIS, have not been comprehensively studied in Nigeria. Thus, this study was limited to answering the basic question of physical availability of and geographical accessibility to accredited healthcare facilities under the NHIS. This would enable a novel opportunity to assess the physical access function of achieving UHC. It would also provide invaluable support for evidence-based planning and decision making on resource allocation in Nigeria and other similar settings. The findings will be useful in taking appropriate steps to reduce physical inequity of access to available healthcare facilities and, by proxy, access to quality healthcare where such is available. The results would be appropriate in other settings planning to implement similar reforms for UHC.

## Materials and Methods

### Study design and area

This was a multidisciplinary study with a descriptive cross-sectional component and a geospatial mapping. It was conducted in the 11 local government areas (LGAs) of Ibadan, Oyo State, Nigeria. The 11 LGAs are made up of 5 urban and 6 semiurban components. The semiurban LGAs form an outer ring of the inner five LGAs.^12^ Currently, the population of the 11 LGAs is about 3 million, based on a projection using the figure from the 2006 Nigeria population census as the base year.^13^ There were several healthcare facilities at the primary, secondary and tertiary care levels in the study area. However, it is important to note that the NHIS did not engage the PHC facility level to provide care for its enrollees. Provision of care under the scheme was limited to secondary and tertiary care level.

### Data sources

A list of accredited facilities was obtained from the NHIS Oyo State Office, Ibadan. This was corroborated with the list that was obtained from the NHIS zonal office to ensure reliability. In this study, satisfaction with care provided in the healthcare facilities is the main outcome variable. The proportion of enrollees who were satisfied with choice of provider (a domain of measure of responsiveness or health system efficiency and performance) in a similar and recent study in Nigeria was 40.7%.^14^ Using the Leslie Kish formula,^15^ the calculated minimum sample size was 420. Eligible individuals were the principal enrollees or spouses (excluding dependents aged <18 y) and had enrolled in the facility for at least 1 y prior to the commencement of the study. Selected enrollees (n=420) in the 11 LGAs were interviewed in selected NHIS-accredited health facilities with the aid of a semistructured interviewer-administered questionnaire. During the interview, the name of the nearest bus stop or a major landmark to the places of residence of study participants were obtained. This approach was adopted to ensure and maintain the confidentiality of the study participants, which could easily have been breached were the real residential addresses were obtained and used.

### Sampling strategy

A list of all healthcare facilities within the study area (11 LGAs), primary, secondary and tertiary care level facilities, was obtained from the Oyo State Ministry of Health. Coordinates of all facilities, both NHIS-accredited and others that were not engaged by the NHIS, were determined using Global Positioning System (GPS). The location of all facilities was recorded using GPS and subsequently mapped using ArcGIS software. Next, a list of all NHIS-accredited facilities within the study area was obtained from the NHIS Oyo State Office in Ibadan. As for the choice of enrollees, 11 NHIS-accredited health facilities, and 1 facility in each of the 11 LGAs, was selected by simple random sampling to make a total of 11 facilities (selected across 11 LGAs). The selected facilities were visited and the number of enrollees in each of the selected health facilities was verified. Proportionate allocation of the estimated sample size (420) was performed based on the number of enrollees across the selected NHIS-accredited facilities.

### Participant selection and GPS mapping

A list of NHIS enrollees waiting to receive care in the outpatient section of a selected health facility was obtained from the medical records department of the facility. Eligible individuals were the principal enrollees or spouses (excluding dependents aged <18 y) and had enrolled in the facility for at least 1 y prior to the commencement of the study. This was to ensure that study participants had adequate knowledge of the basics of the NHIS, and enough interaction with the health system under the scheme that enabled them to respond appropriately.^14^ Among this population, enrollees who began using the selected facilities before the commencement of the health insurance scheme were excluded from the study, as well as enrollees who were healthcare workers in the selected facilities. A sampling frame was generated, the sampling interval was determined and systematic random sampling was used to select eligible participants. Systematic sampling was chosen because it eliminates the phenomenon of clustered selection and a low probability of data contamination. The disadvantage of using a systematic sampling technique was noted and is considered as a study limitation. Hospital card numbers of enrollees who were interviewed were documented and kept safe. Individuals (enrollees) who had earlier been interviewed during the study but came back to the clinic for care were deliberately identified and excluded. This was done so as not to interview such individuals a second time. This was carried out by crosschecking the hospital number of the prospective interviewee (enrollee) against the list of hospital numbers that were documented earlier for safekeeping. This exercise was repeated daily until the whole sample was interviewed. The residential address (represented by the name of a major landmark, e.g. the closest bus stop or any other major landmark) of each enrollee interviewed was obtained.

### Geospatial data analysis

#### Mapping the NHIS-accredited facilities

To determine the distributional pattern of NHIS facilities in Ibadan, the Nearest Neighbour (Rn) Statistic was algorithm implemented in ArcGIS software.^16^ The algorithm provides an objective mathematical description of spatial point events in space. In this regard, it describes the spatial arrangement of both the NHIS-accredited and non-accredited facilities in Ibadan in terms of whether they are clustered, random or regular. The analysis was computed at 5.0% significance level. For the NHIS-accredited facilities, whatever distribution pattern emerges from the analysis has implications for enrollee accessibility and utilisation of services provided by NHIS facilities.

#### Identification of enrollees’ nearest NHIS facility

The identification of enrollees’ nearest NHIS facility was accomplished using the GPS locations of all accredited NHIS facilities in the metropolis. In addition, the approximate coordinates of all the respondents interviewed in this study were also obtained. Residential coordinates of the respondents could not be directly obtained through Open Data Kit Collect because respondents were interviewed outside of their residence. Hence, an accurate description of their residence was requested, with a view to using other methods to estimate their residential coordinates. Each enrollee’s street address was searched for on Google Earth satellite image and the search was enhanced by field knowledge of the names of the bus stops, major landmarks and streets closest to the places of residence of enrollees (obtained during the interview). Such locations were extracted from Google Earth as a single x and y coordinate.

Similarly, the locations of healthcare facilities utilised by respondents (NHIS enrollee as indicated in the questionnaire) were extracted from the list of NHIS-accredited facilities in the metropolis. Coordinates of the facilities were obtained during client interviews at those facilities. Therefore, these two data layers—enrollee’s residence (closest major landmark as proxy for residence) and location of healthcare facilities typically used by enrollees—were used in the spatial analysis to identify the closest NHIS-accredited healthcare facility to each enrollee residence and to also estimate the distance between an enrollee’s location and each NHIS facility being utilised. The ‘Distance to the Nearest Hub (points)’ function in Quantum GIS (QGIS) V. 3.10 was used to automatically assign enrollees to the nearest NHIS facility while the ‘Join by lines (Hub Lines)’ functions were used to assign enrollees to the NHIS facility they use. These two functions in QGIS V. 3.10 provide not only the maps showing the connectivity, but also information about distances between enrollees and their nearest NHIS facility, as well as the distance between enrollees and the NHIS facilities they have been using.

### Methods and discussion for nearest neighbor analysis

The spatial pattern of health facilities in Ibadan was analysed using the nearest neighbour analysis (NNA) statistical algorithm in Arc GIS 10.4 software. A GPS device was used to determine and record the locations (coordinates) of accredited NHIS and other health facilities in the study area. The coordinates were subsequently imported to the ArcGIS software, where they were mapped and overlaid on the administrative map of the Ibadan metropolis. It should be noted that political boundaries of the study area were also digitised from a georeferenced map of the Ibadan region using a projected coordinate system (UTM 31N).

**Figure 1.**
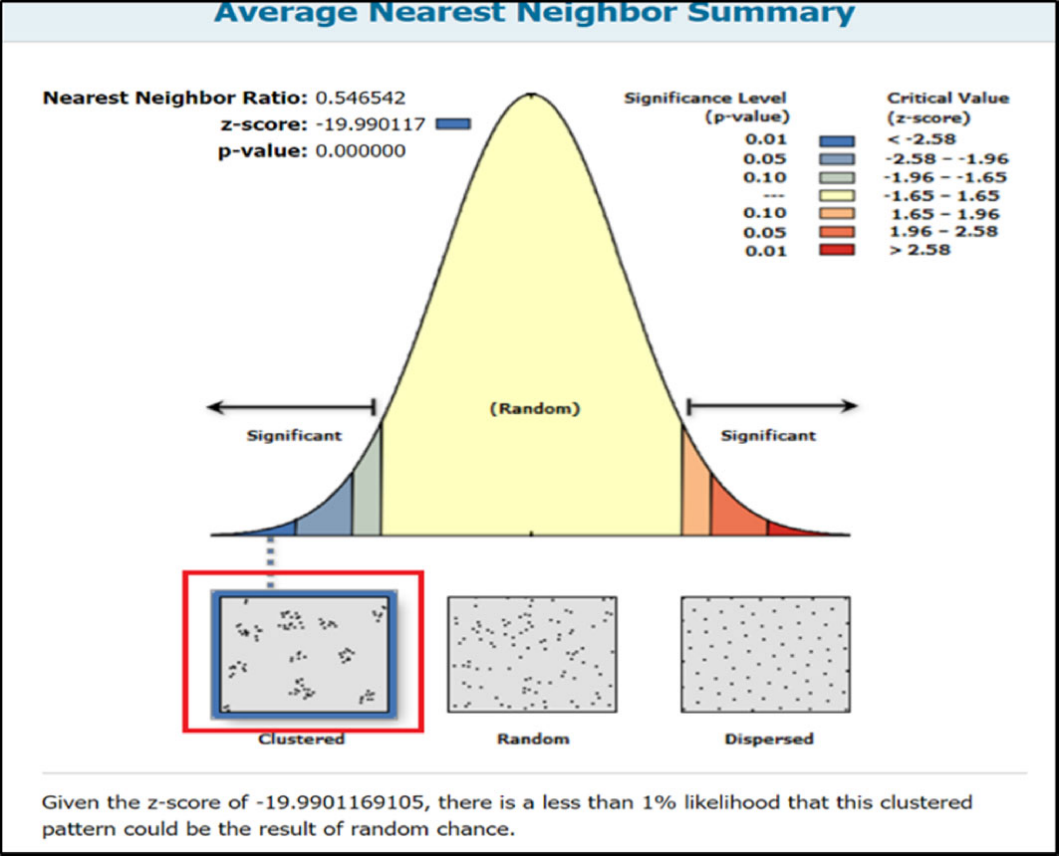
Clustering analysis of accredited health facilities.

The coordinates of the health facilities, as well as the shapefiles of the study area, were saved in the same projected coordinate system (UTM Zone 31 N) for better analytical results. The average NNA was used to analyze the spatial pattern of distribution of the healthcare facilities. The result shows that the spatial pattern of health facilities distribution in the study area is clustered (Figure 1). Thus, given the z-score of 9.990117, there is a less than 1% likelihood that this clustered pattern could be the result of random chance. The result of the analysis shows that Rn 0.842974 (Rn<1) was obtained at a z-score of 9.990117 (Table 1). Hence, there is significant clustering of healthcare facilities in some localities in Ibadan, resulting in unequal access to NHIS-accredited facilities in the metropolis.

### Cluster analysis of health facilities in Ibadan

**Figure 2.**
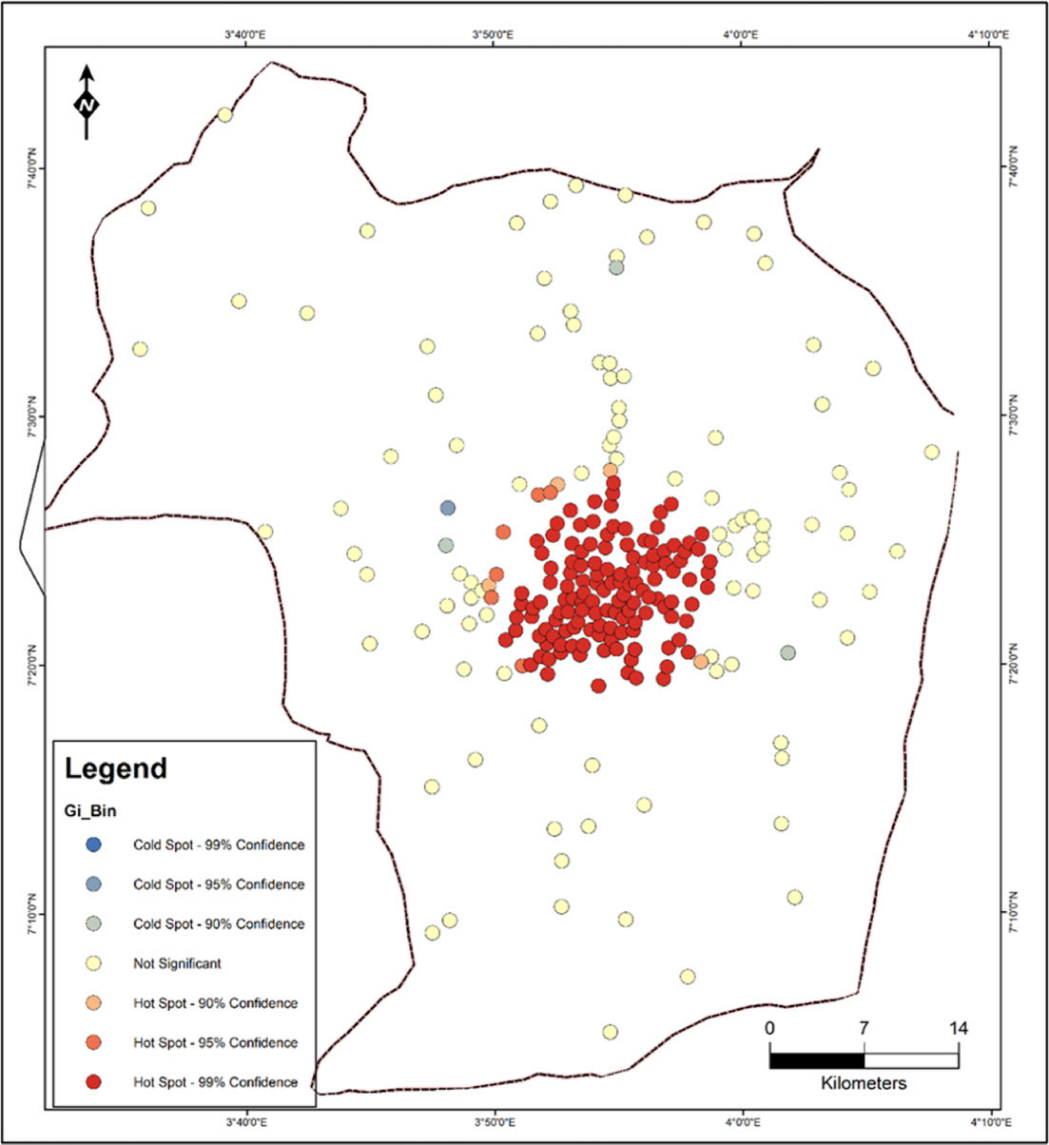
Spatial pattern of health facilities in the study area.

To further show the degree of clustering of the result, the cluster analysis ‘optimised hot spot’ tool in ArcGIS V. 10.4.1 was used. Hotspot analysis was used to identify neighbourhoods/localities with the clustering. The resultant Z-score is used to identify whether a neighbourhood can be characterised as a hotspot or a cold spot. A high z-score and a low p value for a feature indicates a significant hotspot, while a low negative z*-*score and small p value indicate a significant cold spot. The higher (or lower) the z-score, the more intense the clustering. A z-score near 0 indicated no spatial clustering. The result of the analysis of health facilities shows that there is a statistically significant hotspot of health facilities at 99% confidence located around the urban areas of Ibadan. The significant hotspot result is dominated by a feature with a high value and is surrounded by other features with high values as well. Away from the urban built-up area of Ibadan, health facility clustering is not statistically significant (See Figure 2).

## Results

Table 2 shows the population of urban and semiurban (rural) LGAs. The urban LGAs' population was 1889776 (53.4%) of the total population in the 11 LGAs. It should be noted that the total number of healthcare facilities in the urban LGAs was higher only by 18 (3.1%) than it was in the semiurban LGAs. Population coverage under the NHIS was 103 313 (5.5%) of the total population (1889 776) of urban LGAs, while it was 8808 (0.5%) of the total semiurban population (1648 675). Of the total 112 121 enrollees accessing care under the NHIS, the urban LGA population comprised 103 313 (92.1%), while 8808 (7.9%) were in the semiurban LGAs. It is important to note that the NHIS did not accredit PHC facilities to provide care under the scheme.

**Table 1.** Statistics of nearest neighbour analysis of health facilities in Ibadan

Facility	No. of facilities	Z-score	p-value	Observed mean distance (m)	Expected mean distance (m)	Rn-value	Area of study (m^2^)	Pattern
Health facility	531	-9.990117	<0.001	670.6474	1227.0741	0.842974	3198 130 000.0	Clustered

**Source:** Author's analysis 2020.

**Table 2.** Distribution of NHIS-accredited health facilities and enrollees across LGAs in Ibadan

				Total facility				NHIS status	
S/N	LGAs and location urban		Population @ 2.7% annual GR	1^0^	2^0^	3^0^	Total facility in LGA	No. of NHIS facilities	Total enrollees in LGA
1	IBN		856 988	24	63 (37*)	1 (1*)	88	38 (43%)	42 429
2	IBNE		330 399	13	36 (31*)	0	49	31 (63%)	12 792
3	IBNW		152 834	9	27 (22*)	0	36	22 (61%)	23 593
4	IBSE		266 457	15	14 (7*)	0	29	7 (24%)	769
5	IBSW		283 098	23	79 (65*)	0	102	65 (64%)	23 730
Suburban			1889 776				304		103 313
6	Akinyele		211 359	36	10 (3*)	0	46	3 (7%)	204
7	Egbeda		319 388	34	42 (9*)	0	76	9 (12%)	6857
8	Ido		117 129	18	27 (11*)	0	45	11 (24%)	1188
9	Lagelu		147 957	19	10 (6) *	0	29	6 (21%)	559
10	Oluyole		734 377	26	12	0	37	0 (0%)	0
11	Ona-Ara		118 465	29	24	0	53	0 (0%)	0
	Total		1648 675				286		8808
	Gross total		3538 451						112 121

Abbreviations: GR, growth rate; IBN, Ibadan North; IBNE, Ibadan Northeast; IBNW, Ibadan Northwest; IBSE, Ibadan Southeast; IBSW, Ibadan Southwest; 1^0^ (Primary Health Care Facility); 2^0 (^Secondary Health Care Facility); 3^0^ (Tertiary Health Care Facility); SU, semiurban.

*NHIS-accredited healthcare facilities (e.g. in IBN, of the 63 secondary healthcare facilities, 37 were NHIS-accredited facilities).

Table 3 shows the NHIS-accredited facilities and the bypassing rate for each of them. Overall, >90% of all study participants did bypass nearby health facilities to receive care. The average distance covered by an individual study participant was 1.096–5.914 km (Table 4).

**Table 3.** Health facility bypassing status of enrollees

		Bypassed		Non-bypassing		
Serial no.	Patronised health facility	No.	%	No.	%	Total
1	Teju	59	98.3	1	1.7	60
2	St. Mary	59	100	0	0.0	59
3	St. Marello	29	100	0	0.0	29
4	St. Dominic	30	81.1	7	18.9	37
5	Cottage Police Clinic	39	95.1	2	4.9	41
6	Lafia	63	100	0	0.0	63
7	LAD	39	81.1	9	18.9	48
8	Jericho	14	100	0	0.0	14
9	Immaculate	50	100	0	0.0	50
10	Doctor's Polyclinic	10	66.7	5	33.3	15
11	Chrisbo	13	86.7	2	13.3	15
Total		405	94.0	26	6.0	431

**Table 4.** Average distance travelled by enrollees bypassing health facilities

		Average distance travelled	
Serial no.	Hospital	Shortest distance (km)	Longest distance (km)
1	Chrisbo	1.095	2.732
2	Doctors Polyclinic	1.021	3.731
3	Immaculate	1.141	7.617
4	Jericho	0.763	3.805
5	Lad	0.513	6.062
6	Lafia	1.157	7.329
7	Police Cottage	0.833	5.709
8	St. Marello Catholic	1.932	8.595
9	St. Mary Catholic	1.752	7.234
10	St. Dominic	0.978	6.156
11	Teju	0.866	6.089
	Total average	1.096	5.914

**Figure 3.**
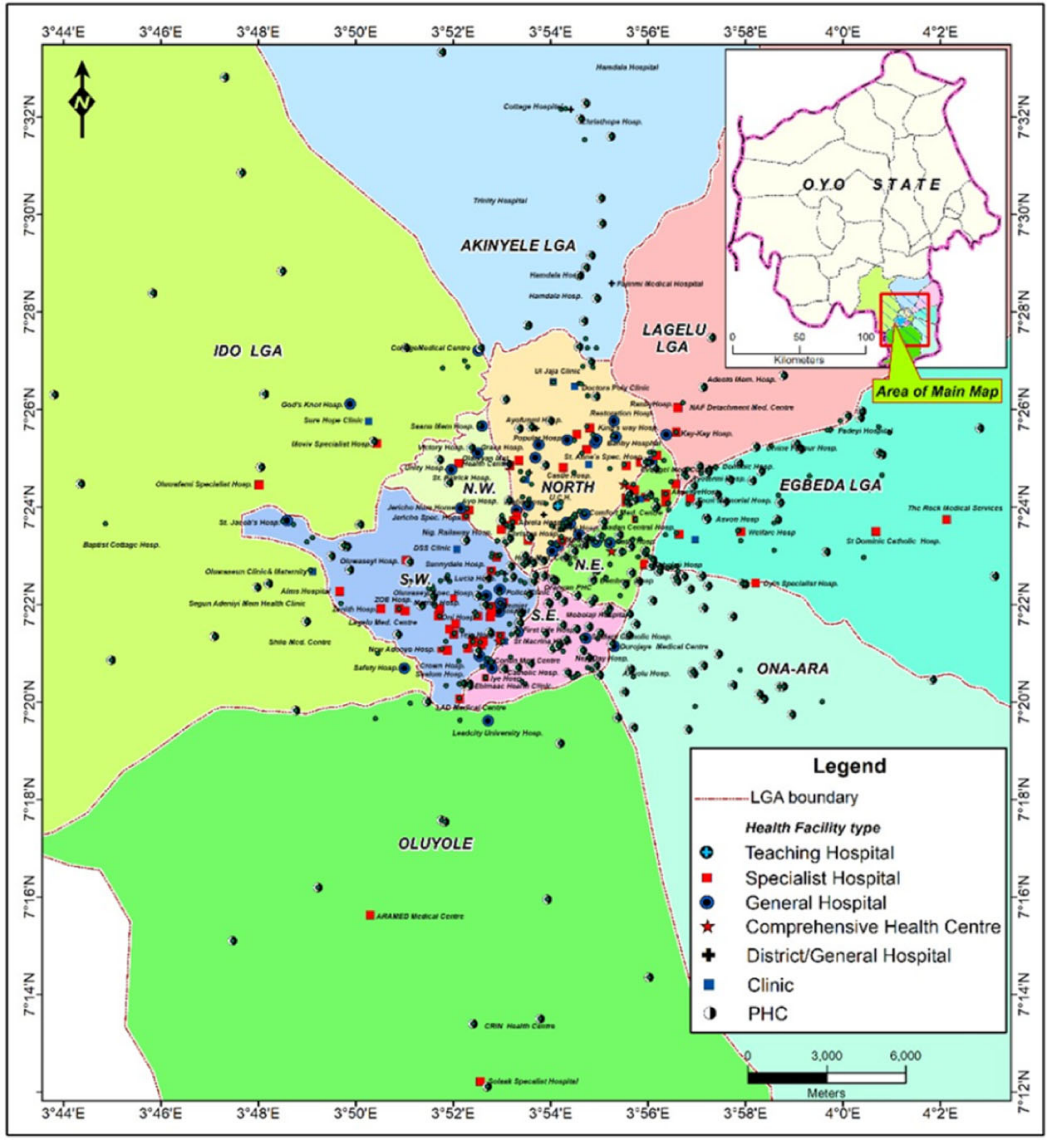
Types of health facilities in the study area.

Figure 3 shows the geospatial relationship between the 11 LGAs that constitute the study area. It also shows various types of healthcare facilities providing services at different levels of care, that is, primary, secondary and tertiary. These facilities belong to both the public and private sectors. From Figure 3, it can be seen that most of the facilities are clustered within the centre, which corresponds to the five inner core LGAs of Ibadan, while the peripheral areas of the city, which contain the remaining six LGAs, had few healthcare facilities scattered within them. It should be noted, however, that PHC facilities have a better spread throughout the 11 LGAs and were most prominent in peripheral LGAs compared with secondary and tertiary level facilities.

**Figure 4.**
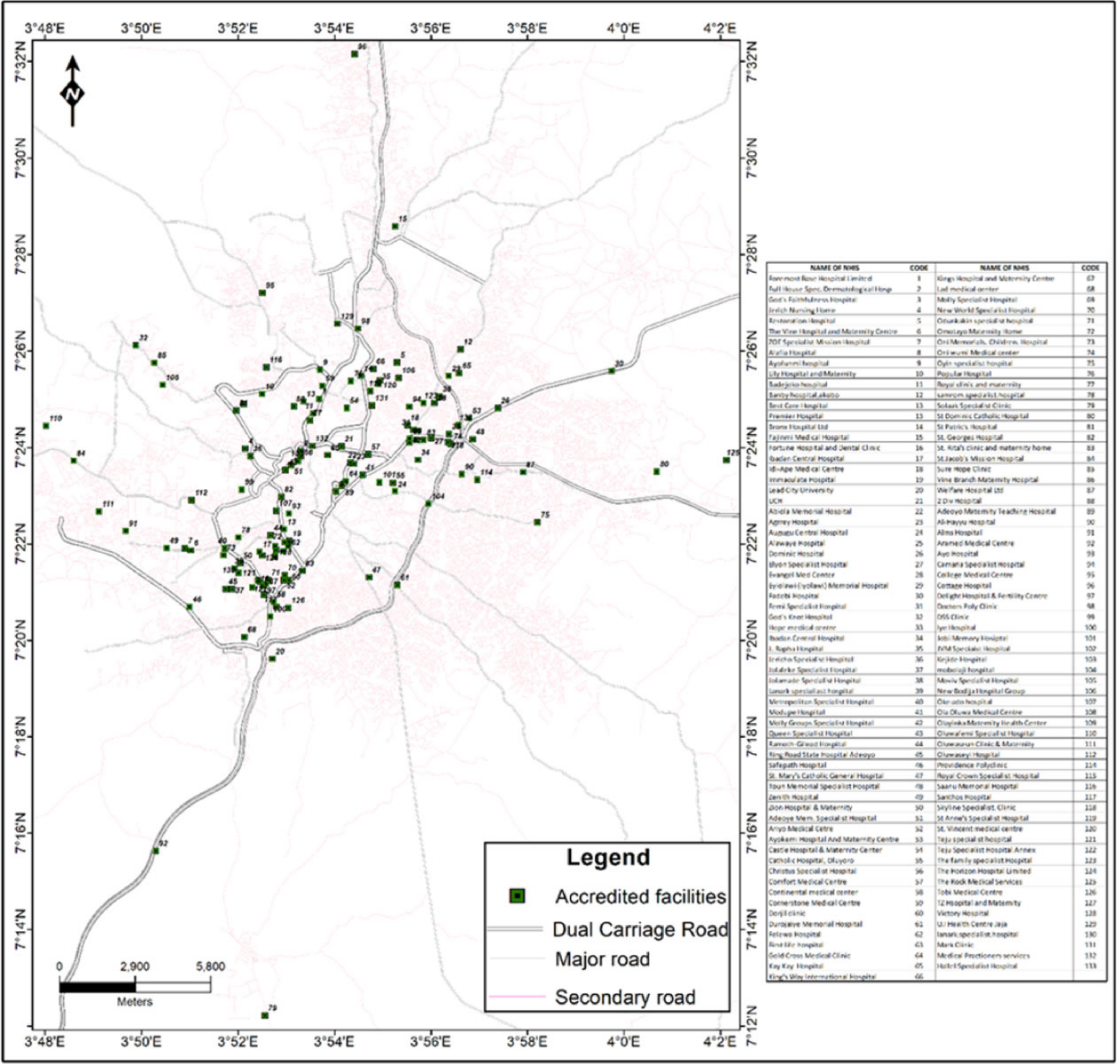
NHIS-accredited facilities in the study area.

Figure 4 shows only the NHIS-accredited facilities in the study area. Similar to the distribution of all other facilities, including those that were not providing services under the NHIS, the majority of these facilities were clustered within the inner five LGAs of Ibadan, while the peripheral parts of the city had sparsely distributed NHIS-accredited health facilities. The same reason as provided above is sufficient to explain this distribution pattern.

**Figure 5.**
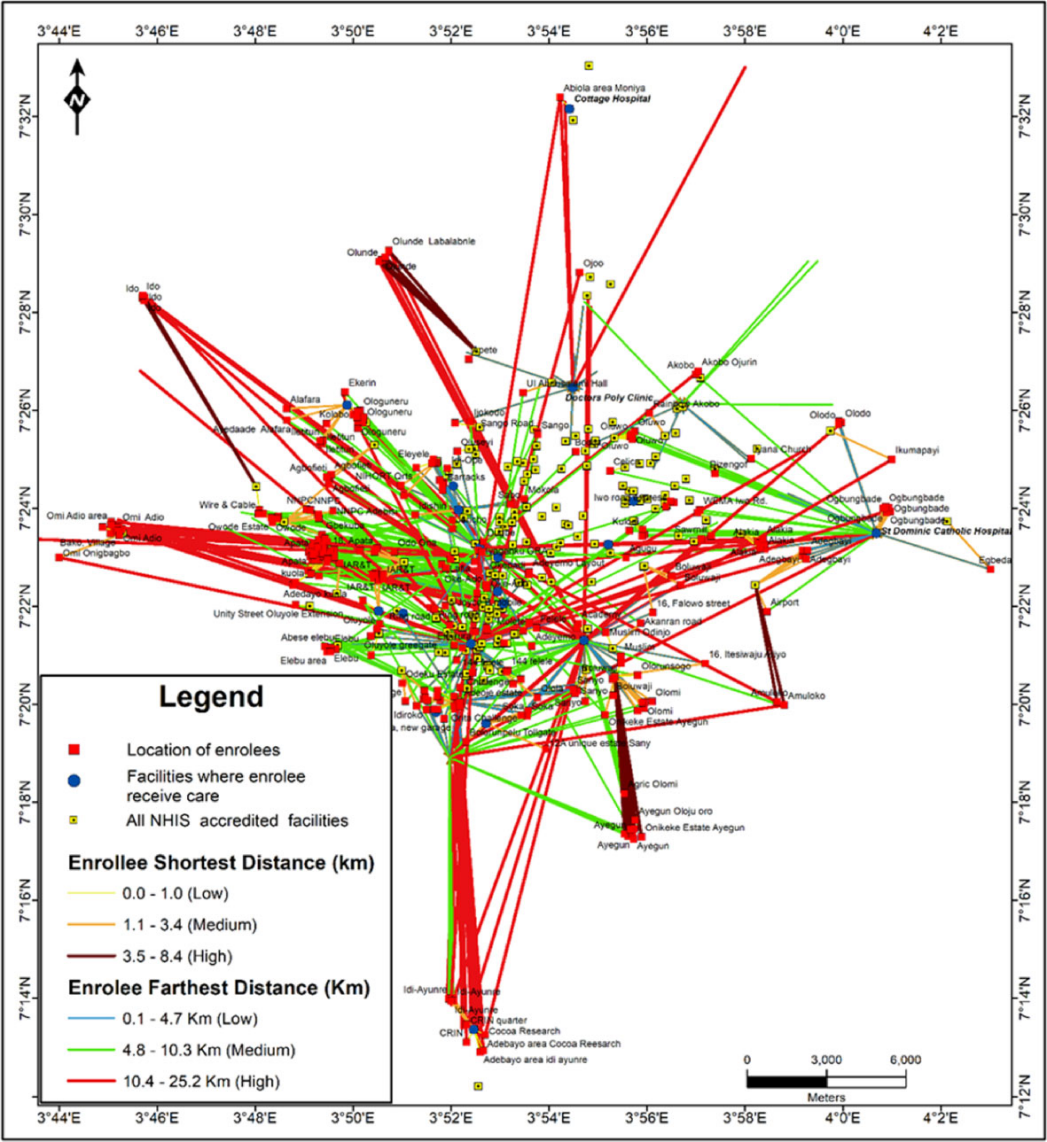
Spatial relationship between enrollees’ residence, closest and furthest NHIS-accredited facilities for care.

Figure 5 is a map that shows the geospatial relationship between NHIS-accredited facilities and enrollees’ residences. Enrollees receive healthcare services in accredited facilities. However, while some patronised health facilities that were closest to their residence, the majority did so in health facilities that were further away from where they (i.e. the enrollees) lived, as indicated by the longer straight lines. Also, the majority of enrollees seemed to patronise facilities that were located in the centre of the study area, which corresponds to the five inner LGAs. Evidence of this was demonstrated by the heavy traffic lines towards the centre of the map compared with the thinner lines, which indicate lighter traffic and are thus less travelled.

## Discussion

In this study, we found that most of the healthcare facilities in the study area were clustered within the inner core of Ibadan, while the peripheral areas of the city had few and scattered health facilities. However, the distribution of PHC facilities was more equitable than secondary and tertiary facilities across all the LGAs in the study area. The observed pattern of spread of health facilities in the current study corroborated the pattern found in earlier studies conducted in Nigeria and elsewhere.^17,18^ The pattern of distribution of healthcare facilities within the study area may not be unconnected with the city’s growth, which tends to be centrifugal in nature, with the result that the further away one travels, the sparser healthcare facilities become. This may also have to do with the availability of infrastructure, such as better road networks, as well as population density, which are more likely to be higher in the core inner areas of cities than in periurban areas.

The current study shows that the city of Ibadan contains healthcare facilities at different levels in a centripetal pattern. This suggests that healthcare services are available and could be accessed by those who need them, provided other factors such as geographical access are not constraining issues. While the presence of a social health insurance scheme does not guarantee access to needed services,^19^ the presence of a social health insurance scheme, as is found under the NHIS, has the potential to minimise financial constraint as a factor of inequitable access to available healthcare. Yet access to healthcare in Nigeria is poor and is worse in semiurban and rural areas.^5^ The availability of healthcare facilities and geographical accessibility are some of the factors that determine access to healthcare services. It should be noted that PHC facilities were not engaged by the NHIS to provide services to enrollees, yet PHC facilities had the most equitable geospatial spread of the three levels of care, as was demonstrated in the maps. The Nigerian government has adopted the PHC level as the entry point into the health system. For this, the number of PHC facilities in a LGA was determined by the number of administrative wards in that LGA. As such, PHC facilities are widespread in both rural and urban areas, constituting >85% of all healthcare facilities at the rate of almost one PHC facility per ward throughout the country.^11^ From this picture, achieving UHC would have been more equitable if PHC facilities were engaged as service providers. Countries with remarkable achievement towards UHC have been reported to engage the first level of care of the health system in service provision. In Rwanda, Ghana and Brazil, PHC facilities were engaged as service providers. The involvement of the lowest level of care in addition to higher levels contributed to the successful health reform for UHC in these countries.^20,21^ In Nigeria, however, despite the availability of widespread PHC facilities in both urban and rural areas, these centres were not utilised as service providers under the NHIS.

Grossly, the distribution of total healthcare facilities in the study area was skewed in favour of urban settlement, even although the population difference between the two parts of the city was not substantial. In a similar manner, population coverage by the NHIS in the urban area was much better than it was in the semiurban settlement.^9^ This is despite closer proximity of semiurban settlement to the metropolis. The situation is worse in the remote rural areas.^22^ It is not clear how the country intends to achieve UHC with the current pattern of distribution of health facilities and the non-involvement of the PHC centres as service points. What is clear, however, is that countries that have had encouraging performance on the way to achieving UHC made use of the lowest levels of care, which are incidentally the closest to service users. The literature shows an association between the distance to receive care and adverse health outcomes. Small distances from health facilities were associated with a higher likelihood of child mortality. It also increased the chances of non-use of health facilities.^2^ An association between long distances to health facilities and poor health outcomes has been reported among victims of snakebite in Nigeria.^8^ Distance between residence and health facilities has been shown to shape healthcare-seeking behaviour, as the longer the distance then the less likely people are to patronise such facilities and the more likely they are to adopt unsafe alternative care.^5^ Non-utilisation or a delayed use of health services with increase in distance between residence and health facilities have been reported in much earlier studies in Nigeria,^23,24^ with consequently poor health outcomes.^25^ However, the clustering of most of these facilities in the centre of the city is suggestive of better access to healthcare among those who reside in the urban area than among those who reside in semiurban and rural areas of the city. However, it has been shown that proximity to a healthcare facility does not necessarily translate to better health facility patronage.^19^

When individuals patronise a facility that is further away instead of the facility with the lowest travel cost, this phenomenon is called bypassing.^23^ In our study there is evidence of the degree of bypassing of NHIS-accredited facilities among NHIS enrollees. The geospatial pattern and distribution of the facilities chosen for care, and a numerical equivalent (and proportion) of those who bypassed the closest health facility to receive care in the selected study facilities, are evidence of bypassing in the study area. Overall, the rate of bypassing varied across facilities. The majority of those who bypassed seemed to be unaware of this. This tends to support the hypothesis that study participants may not be aware of closer facilities to their residences.

The average distance travelled to access care was 1–6 km. Many factors have been put forward for the bypassing phenomenon. Previous studies that have studied bypassing of health facilities^26,27^ have portrayed the phenomenon as a simple deliberate action on the part of healthcare consumers seeking a better quality health service. Inadequate knowledge of clients of the availability of closer health facilities is another likely reason. Although this factor was not explored, stigmatising illnesses such as TB and HIV/AIDS could lead to individuals preferring a facility further away from home rather than one that is closer.^28^ Other factors that have been cited as probably responsible for bypassing could be exigencies such as the location of healthcare facilities close to workplaces, frequently patronised areas such as marketplaces, as well as residences of family members and friends.^29^ This study claims that the phenomenon of bypassing actually took place in this study population. This claim is reinforced by the reason that almost all (94.0%) of the study population did actually bypass, that is, it was too large a number for a non-routine event such as referral to account for. This is more so that the process of selection of study population employed probability sampling methods. However, irrespective of the reasons behind the (bypassing) phenomenon, travelling long distances to access care may result in poor health outcomes.^2^ Thus, the government and other stakeholders must endeavour to address the responsible factors as appropriate.

Euclidean metric (straight-line distance between two points) was used as proxy for spatial access to health service points (facilities). It is noteworthy that this method does not take into consideration topographic and infrastructural barriers such as elevations, slopes, water bodies and other physical barriers on the way to accessing healthcare in facilities. It is acknowledged that these barriers could be taken care of by other methods such as the network analysis method for distance measurement. However, it should be noted that Euclidean measures are acceptable, considering the cost and time in resource-poor settings, where travel is largely done by walking through largely non-motorised pathways, and where there is a lack of actual travel time and cost data and self-reported travel time is usually inaccurate.^30^ The scope of the current work could not accommodate the reason for bypassing as it was observed in this study. The distance between actual participants’ residential locations and the landmarks used as proxies was 300–500 m. It is acknowledged that using landmarks as proxies for participants’ residential locations could affect the distance measurements. We accept all these as limitations.

### Conclusions

In contrast to developed economies, GIS studies are not common in sub-Saharan Africa, however, they are highly relevant to solving the high burden of diseases and other health-related challenges in the region.^6^ The current study has provided invaluable findings that are not common in health system assessment in this environment, and therefore would be useful for evidence-based, decision-making policies in health planning and efficient allocation of health resources by need in population groups. This will assist in minimising inequity of access to required healthcare resources and services,^31^ which, judging by the findings in this study, is more likely to be worse in the rural areas of Nigeria and in other similar settings.^2,5^

## Data Availability

The datasets used and/or analysed during the current study are available from the corresponding author on reasonable request.
